# Supplementary material for: Identification of photoperiod-induced specific miRNAs in the adrenal glands of Sunite sheep (Ovis aries)
Source: Front Vet Sci. 2022 Jul 22;9:888207. doi: 10.3389/fvets.2022.888207 (PMC9354845; doi:10.3389/fvets.2022.888207)
Supplement: Supplementary file 1 [file Data_Sheet_1.ZIP › Supplementary file/miRDeep2 file.zip!/miRDeep2 file/Novel_Structure/SP42A3/Novel_708.pdf]

Provisional ID	: NC_019460.2_7454
Score total	: 1.7
Score for star read(s)	: -1.3
Score for read counts	: 0
Score for mfe	: 1.4
Score for randfold	: 1.6
Score for cons. seed	:
Total read count	: 32
Mature read count	: 32
Loop read count	: 0
Star read count	: 0

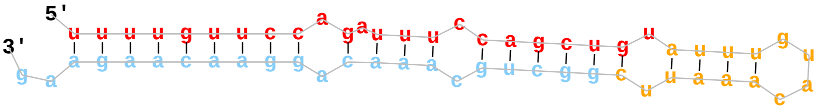

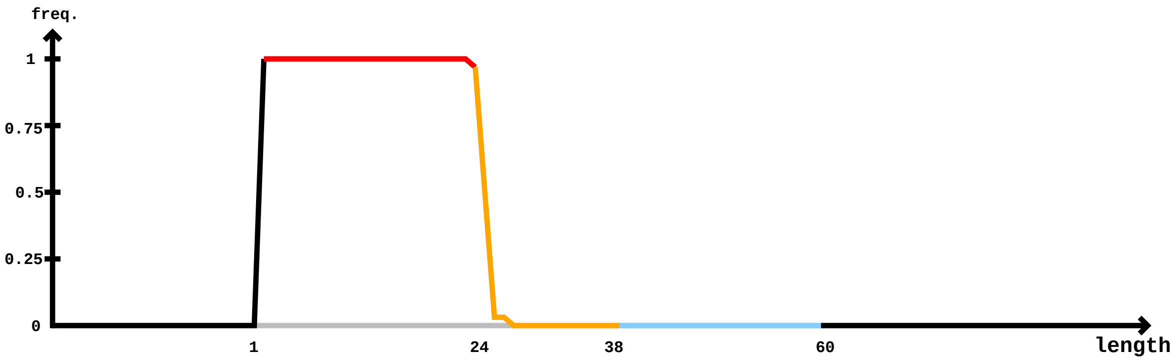


```
                    Mature                    Star
5'- accuuuggaguuggaugacuuuuuguuccagauuuccagcuguauuuguacaaauucggcugcaaacaggaacaagaaguugucgcuuaacucaaguagauccuuggaacug   -3'    exp
    ..((...((((((((((((((((((((((.(.(((.((((((.((((....)))).)))))).)))).))))))))))))))))...))))))....)).............   reads      mm         sample
    ....................uuuuguuccagauuuccagcug......................................................................   1          0          seq
    ....................uuuuguuccagauuuccagcugu.....................................................................   26         0          seq
    ....................uuuuguuccagauuuccagcugua....................................................................   4          0          seq
    ....................uuuuguuccagauuuccagcuguauu..................................................................   1          0          seq
```
